# Comparative Evaluation of Biochemical and Hematological Parameters of Pre-Storage Leukoreduction during RBC Storage

**Published:** 2018-01-01

**Authors:** Behrooz Ghezelbash, Azita Azarkeivan, Ali Akbar Pourfathollah, Mohammadreza Deyhim, Esmerdis Hajati, Alireza Goodarzi

**Affiliations:** 1 Laboratory Hematology and Blood Bank, Blood Transfusion Research Center, High Institute for Research and Education in Transfusion Medicine, Tehran, Iran; 2 Pediatric Hematology Oncology, Iranian Blood Transfusion Research Center, High Institute for Research and Education in Transfusion Medicine, Thalassemia Clinic, Tehran, Iran; 3 Blood Transfusion Research Center, High Institute for Research and Education in Transfusion Medicine, Tehran, Iran; 4 Blood Transfusion Research Center, High Institute for Research and Education in Transfusion Medicine, Biochemistry Department, Tehran, Iran; 5 Laboratory Hematology and Blood Bank, Blood Transfusion Research Center, High Institute for Research and Education in Transfusion Medicine, Flow Cytometry Department, Tehran, Iran

**Keywords:** Leuko-reduction RBC, Blood transfusion, RBC storage, RBC storage lesion

## Abstract

**Background: **Some of the red cell storage lesions (RCSLs) take place during red blood cell (RBC) storage and may reduce the function of these cells dramatically, which mostly caused by residual leucocytes in blood components. This study was planned to observe the biochemical and hematological changes in pre-storage leukoreduced RBC (LR-RBC) compared with unfiltered RBC during *in vitro* storage.

**Materials and Methods: **Ten unit RBCs were collected, processed and stored according to Iranian standard operating procedure (SOP) of Iranian Blood Transfusion Organization (IBTO). Every unit was split into two equal parts, unfiltered RBC and LR-RBC. Samples were collected and tested on weeks of storage. Biochemical parameters such as lactate dehydrogenase (LDH), lactate concentration and glucose-6-phosphate dehydrogenase (G6PD) enzyme activity were measured by auto-analyzer. In addition, hematology analyzer was used to monitor the change of RBC indices such as (MCV), (MCH) and (MCHC).

**Results: **In this study, both groups showed progressive increase of LDH and lactate levels, and also G6PD activity decreased during storage. Mean of LDH and lactate in unfiltered RBC was significantly increased compared with LR-RBC during all days of storage (*p*< 0.05). There was statically significant decrease in the G6PD enzyme activity between the two groups and weeks of storage (*p*< 0.05). However, the RBC indices remained within the expected levels in both groups.

**Conclusion: **LR-RBC and RBC both exhibited RCSL during storage, but LR-RBC is effective in reducing Red cell storage lesion (RCSL)** and **also improves the quality of stored red blood cells.

## Introduction

 Leuko-reduction or leuko-depletion refers to a reduction in the number of leucocytes less than 5 × 10^6^ residual donor leucocytes in final blood product while maintaining 85% of viable original RBC^1^. The presence of leukocytes in blood components is responsible for several complications. Leuko-reduction prevents febrile reactions, the accumulation of cytokines/chemokines, avoiding febrile non- hemolytic transfusion reaction (FNHTR), reducing the transmission of cytomegalovirus, and refractory to platelet transfusion (in platelet concentrates) ^2^.

Despite the use of additive solutions, changes in RBC morphology and metabolism are expected during the storage of blood bags with and without leuko-reduction. Storage of erythrocytes causes some complex structural and biochemical alterations, which are called red cell storage lesion (RCSL)^3^. 

Biochemical alterations include the decrease in glucose 6-phosphate dehydrogenase (G6PD) enzyme activity as an antioxidant enzyme , an increase in vesiculation of the RBC, RBC membrane loss, RBC membrane lysis, reduced levels of 2,3-diphosphoglycerate (2,3-DPG), adenosine triphosphate (ATP), and decreased glutathione reductase (GSH) levels. These processes are accompanied by decreased pH, increased LDH enzyme activity and lactate concentration^4^.

These changes lead to reduced function and survival of RBCs following transfusion^5^. G6PD is an important enzyme in RBC metabolism and the key enzyme of the oxidative pentose phosphate pathway (PPP). In the PPP, nicotinamide adenine dinucleotide phosphate (NADP) is converted into its reduced form, NADPH, which is essential for GSH-mediated protection against oxidative stress^6^ maintaining the red cell integrity.

Also, there are some biochemical changes of RBC concentrates in anaerobic glycolysis. In particular, there is an increase in potassium (K^+^) and lactate levels and a simultaneous decrease in pH, glucose and sodium (Na^+^) levels. The storage time has no impact on calcium (Ca^++^) levels in the RBC concentrates^7^.

The enzyme activity of LDH and K^+^ in erythrocytes is substantially greater (20-160 times) than in plasma and hemolysis may be expected to cause increases these analytes^8^.

The objective of this study was to evaluate the effect of leuko-reduction on the biochemical and hematological parameters of RBC during days of storage and to evaluate the biochemical and hematological changes in pre-storage LR-RBC in comparison with unfiltered RBC.

## MATERIALS AND METHODS

 The study was reviewed and approved by the Institutional Ethics Committee. Screening of donors was conducted according to Iranian National Blood Centre Guidelines (SOPs). Whole blood was selected by random assignment for inclusion in the study. We used a sample size similar to those used in previous studies ^4^, ^15^, and all units were collected from 10 healthy male donors [age 40 ± 12 (mean ± S.D)].

**Preparation of RBC**

Approximately 450 mL of whole blood were collected in standard Quadruple containers (Fresenius Kabi Medicare, Hamburg, Germany) with 63 mL CPD (citrate, phosphate, dextrose) as an anticoagulant from ten male donors. Before separation of blood components, the units of blood were pre-stored for 2-6 h at 22 ± 2°C. Whole blood was centrifuged at 4000×*g* for 8 min at 4°C in a blood bag centrifuge, RotoSilenta 630 RS (Hettich, Tubingen, Germany), and separated into RBC and plasma followed by automatic blood component separation by using blood Separation Press (JMS Dualpress, Germany). The supernatants (plasma) were transferred to the other satellite bag.

Packed RBCs left in the primary bag (approximately 200 mL), and were resuspended in 100 mL of saline, adenine, glucose and mannitol (SAGM) as a preservative solution. Afterwards, RBC units (mean volume 300 mL) were split into two equal units, and therefore about 150 mL of RBCs were passed through the filtration set into another satellite bag (LR-RBC) under sterile conditions. Remaining RBCs in the primary bag were considered as unfiltered-RBC. After gentle mixing under a class II laminar airflow cabinet, 8 mL samples were removed aseptically from each unit on days of storage 1, 14, 21, 28, 35 and 42. On days 1, 14, 21, 28, 35 and 42, 4 mL of the samples were analyzed for hematological parameters and for measuring G6PDenzyme activity. Briefly, 4 mL aliquots of the samples were centrifuged at 4000×*g* for 10 min and supernatant plasma was used for the analysis of biochemical parameters. All samples were analyzed on the day of collection. For this study, RBC and LR-RBC units were stored at 2–6°C for 42 days.

The enzyme activity of LDH was estimated by DGKC method in Hitachi 911 Automatic chemistry analyzer (Hitachi, Japan). Plasma lactate was measured by an enzymatic reaction in Cobas Mira chemistry analyzer (Cobas, Roche Diagnostics, Germany).

Hemolysis rate was evaluated by the absorption spectrum of free hemoglobin (HbO_2_) in 415 nm, 450 nm and 700 nm by using UV/VIS spectrophotometer (CLCIL CE7200, England). The percentage of hemolysis was calculated according to guide to the preparation, use and quality assurance of blood component.

Plasma Hb(mg/dl)=( 154.7×A_415_-130.7×A_450_-123.9×A_700_)

Hemolysis Index % =$\frac{\mathrm{Plasma Hb}\left(\frac{\mathrm{mg}}{\mathrm{dl}}\right)\times 100-\mathrm{HCT}}{\mathrm{HbofBag}(\frac{\mathrm{mg}}{\mathrm{dl}})}$

Quantitative measurement of G6PD enzyme activity was done by enzymatic reaction according to kit protocol (Manufacturer Biolabo SA, Maizy, France) by Hitachi 911 Automatic analyzer (Hitachi, Japan).

In order to determine the concentration of hemoglobin (Hb in g/dL), 0.2 mL of homogenized blood was washed 3 times with 2 mL of saline solution (0.9 g/dL). Tube was centrifuged between each washing step, and the supernatant was removed carefully to avoid the elimination of erythrocytes. After the last washing, the washed erythrocytes were suspended in 0.9 mL of hemolysing solution, let the suspension stand for 15 minutes at 2-8°C, and were centrifuged again. The supernatants were used within 1 hour. Results were calculated in units per gram of hemoglobin (IU/g). 

G6PD enzyme activity (IU/gHb) =$\frac{\left(\frac{\mathrm{\Delta Abs}}{\min}\times 5000\right)}{\mathrm{Hb expressed in g}/\mathrm{dL}}$

Hematological parameters such MCV, MCH and MCHC were measured by automated hematology analyzer (Sysmex KX-21, Cobe, Japan).

**Statistical analysis**

All data were presented as means ± SE of triplicate determinants. Data were analyzed using an unpaired two-tailed *t* test or χ^2^ test. Statistical significances were defined at *P < 0.05, **P < 0.01, and ***P < 0.001 compared to the corresponding controls.

## Results

 According to our results, the activity of LDH increased in two groups of RBC (filtered and unfiltered) during the storage, but in unfiltered RBC enzyme activity was more increased compared with LR-RBC. The increment in LDH enzyme activity along the storage period was statistically significant in both groups (*p *< 0.05). The activity of LDH was significantly higher in unfiltered RBC than filtered RBC on the 1^st^, 14^th^, 21^th^, 28^th^, 35^th^ and 42^th^ days (*p* = 0.001) (Table 1).

**Figure 1:**
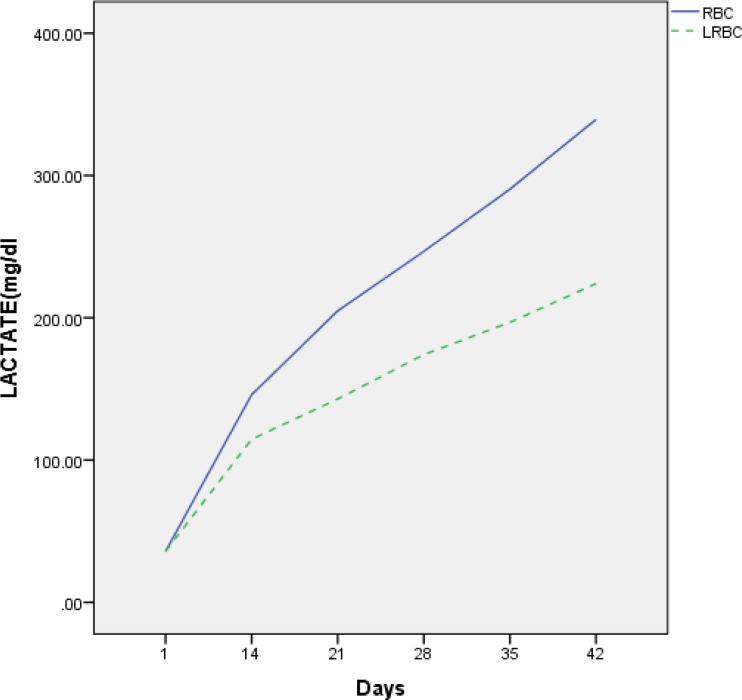
Mean lactate changes in unfiltered RBC compared with LR-RBC during storage.

The lactate concentration was also increased in both RBC and LRBC units throughout the storage period. Statistically significantly higher concentration of lactate was observed in RBC compared to that of LR-RBC on days 21, 28, 35 and 42 (*p* = 0.001) (Table 1). Differences in lactate was observed between the two groups in all steps of testing during storage after day 14 (Figure 1).

**Figure 2:**
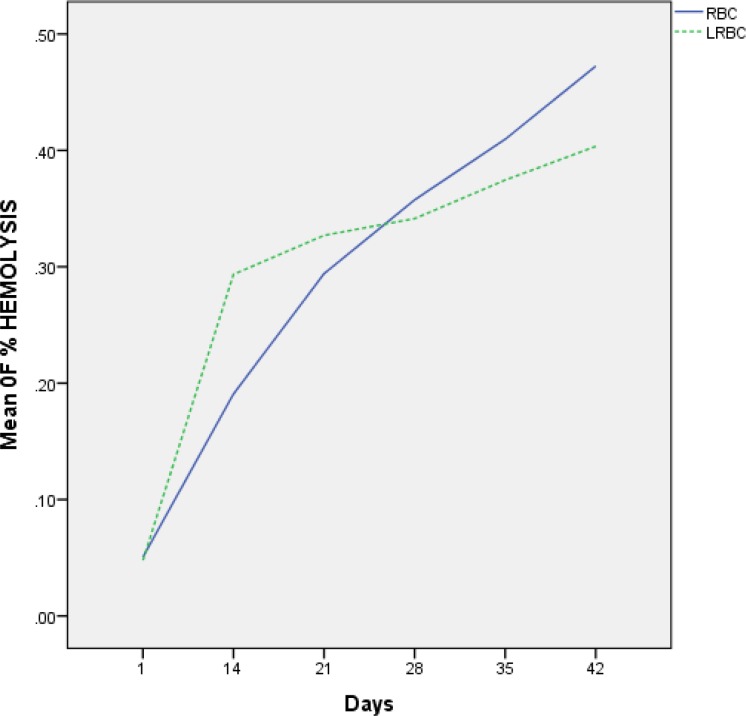
Hemolysis rate in unfiltered RBC compared with LR-RBC during storage.

The rate of hemolysis in the unfiltered RBC and LR-RBC units was increased during storage. The results revealed no significant difference (*p*> 0.05) in percentage hemolysis of the stored blood after 3 weeks, whereas significant increase (p < 0.05) in percentage was recorded after 4 and 5 weeks of storage. On the days of 35 and 42, the hemolysis rate in RBC units was significantly higher than that in LR-RBC (Figure 2). In both groups, hemolysis rate lower than 0.8% was accepted according to the standard range of American Association of Blood Banks (AABB) (Table 1).

**Figure 3:**
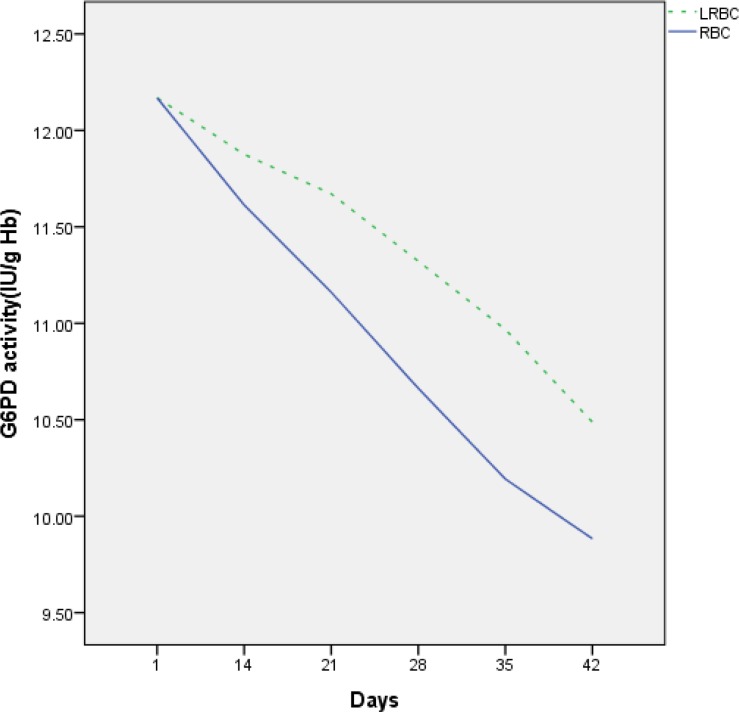
G6PD enzyme activity in unfiltered RBC compared with LR-RBC during storage.

The baseline values of G6PD activities in samples of bags within first day of collection were established. During storage, G6PD enzyme activity decreased in two groups of RBCs (Figure 3). Mean of G6PD activity on day of donation was 12.16±29 IU/gHb in the two groups, whereas gradually decreased to 9.88±0.27 IU/gHb and 10.48±0.36 IU/gHb after 42 days in RBC and LR-RBC, respectively (p = 0.001) (Figure 3). G6PD enzyme activity less decreased in LR-RBC compared with RBC during storage. The results also revealed significant decrease in activity on the last weeks of storage compared to the baseline values (*p* = 0.00) (Table 1).

**Table 1:** Evaluation of biochemical parameters (mean± SE) in unfiltered RBC and LR-RBC during storage

			Day 1	Day 14		Day 21		Day 28		Day 35	Day 42
**LDH(IU/L)**	Unfiltered RBC	126.10±6.46			1265.80±34.89٭٭٭		1497.5±43.16٭٭٭		1671.40±50.72٭٭٭	1836.0±63.66٭٭٭	2105.20±78.62٭٭٭
	LR-RBC	126.10±6.46			287.00±7.31٭٭٭		324.00±6.40٭٭٭		357.20±6.56٭٭٭	387.30±6.02٭٭٭	414.00±6.28٭٭٭
**Lactate ** **(mg/dl)**	Unfiltered RBC	35.86±1.58			146.00±3.81٭٭		204.91±5.32٭٭		246.69±5.77٭٭٭	290.43±5.10٭٭٭	339.40±18.8٭٭٭
	LR-RBC	35.92±1.60			114.70±4.33٭٭		142.97±5.37٭٭		174.14±4.97٭٭٭	196.82±4.48٭٭٭	224.03±6.04٭٭٭
**Hemolysisrat** **e(%)**	Unfiltered RBC	0.0506±0.004			0.2941±0.005٭٭		0.3575±0.015		0.4096±0.012٭	0.54±0.015٭	0.63±0.018٭٭
	LR-RBC	0.0478±0.004			0.3270±0.013٭٭		0.3414±0.015		0.3745±0.015٭	0.461±0.014٭	0.516±0.011٭٭
**G6PD** **(U/g Hb)**	Unfiltered RBC	12.16±0.09			11.61±0.11٭		11.16±0.12٭		10.66±0.16٭٭	10.19±0.10٭٭٭	9.88±0.085٭٭٭
	LR-RBC	12.16±0.09			11.87±0.11٭		11.67±0.086٭		11.32±0.10٭٭	10.96±0.10٭٭٭	10.48±0.11٭٭٭

^@@@^ *p*< 0.01 for overall difference RBC and LR-RBC separately during storage

Results are presented as mean ± SEM, significant differences **between two groups **are indicated by *p< 0.05, **p< 0.01, and ***p< 0.001, and significant differences **one group during storage** are indicated by ^@^*p*< 0.05,^@@^*p*< 0.01, and ^@@@^*p*< 0.001**.**

**Figure 4:**
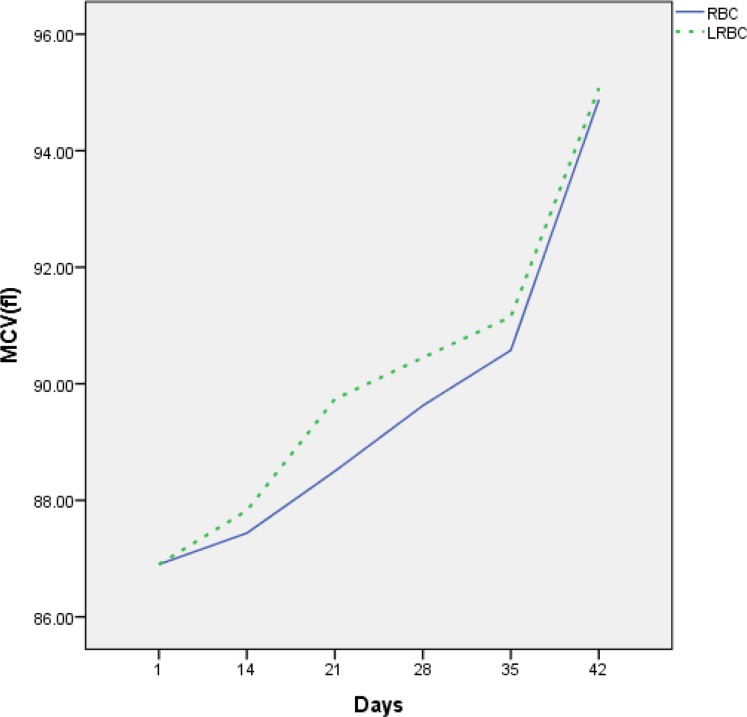
Mean corpuscular volume (MCV) in unfiltered RBC compared with LR-RBC during storage age.

The mean values of some hematological parameters are shown in Table 2. The indices of MCH and MCHC were decreased during storage time, and the amount of MCV was increased in this period (Figure 4). These changes did not cause significant differences between the two groups, while the decrease in MCHC and MCH in one group on day 42 compared to day 35 was significant. MCV shows that there was no significant difference between RBC and LR-RBC, but there was a significant increase in units of RBC and LR-RBC during storage (Table 2).

**Table 2:** Evaluation of hematological parameters (mean± SE) during storage

		Day 1	Day 14		Day 21	Day 28	Day 35	Day 42
**MCV** **(fl)**	UnfilteredRBC	86.90±1.03		87.43±1.04	88.50±1.18	89.62±1.03	90.57±0.99^@@^	94.87±1.10^@@^
	LR-RBC	86.90±1.03		87.82±1.20	89.73±1.07	90.45±1.03	91.15±0.94^@@^	95.07±0.80^@@^
**MCH (pg)**	UnfilteredRBC	28.80±0.53		29.12±0.65	29.20±0.69	29.05±0.68	28.15±0.77^@^	28.89±1.12^@^
	LR-RBC	28.80±0.53		29.12±0.70	29.30±0.55	29.20±0.67	29.22±0.70^@^	28.90±0.59^@^
**MCHC** **(%)**	UnfilteredRBC	33.15±0.20		33.20±0.20	33.00±0.31	32.40±0.31	32.15±0.34^@^	30.42±0.64^@^
	LR-RBC	33.05±0.24		32.57±0.24	32.60±0.28	32.40±0.36	32.07±0.44^@^	30.42±0.38^@^

Results are presented as mean ± SEM, significant differences between two groups are indicated by *p< 0.05, **p< 0.01, and ***p< 0.001, and significant differences in one group during storage are indicated by ^@^*p*< 0.05,^@@^*p*< 0.01, and ^@@@^*p*< 0.001.

## Discussion

 Based on previous studies, during the ex vivo storage of blood components, bioreactive substances accumulated in the storage medium and induced changes in erythrocytes provided permanent evidence for the continuous alterations, resulting in RBCs dysfunction and could have adverse effects on the transfused host^9^^,^^10^. Current laboratory findings are not so perfect and more studies are required in relation to the changes and differences between filtered and unfiltered stored RBC components. Elimination of leukocytes and platelets as a source of free radicals in RBC components has an influence on the protection of oxido-reductive balance in RBC components during storage time. In this study, statistically significant increases in LDH activity were shown along the storage period in unfiltered RBC and LR-RBC. In addition, the enzyme activity of LDH in unfiltered RBC compared to LR-RBC increased from day 14 of storage and was statistically considered significant.

In agreement with the findings of the present study, Verma et al. found a significant increase of LDH on RBC during storage ^11^.  Latham et al. and Bailey et al. also observed an increase in lactate, LDH, and hemoglobin concentration during storage^12^^, ^^13^.

Nedzi et al. reported less increment in LDH (up to 328.2 and 294.2), but, in our study, it was 1836.3 and 387.3 in RBC and LR-RBC, respectively on day 35^14^. This difference could be due to the different storage and assay conditions in the studies. The lactate concentration also increased in both RBC and LRBC units throughout the storage period. Statistically significantly higher concentration of lactate was observed in RBC compared to that of LR-RBC on days 21, 28, 35 and 42 (*p* = 0.001). Differences in lactate were observed between the two groups in all steps of testing during storage after day 14.

The consistency of free Hb and hemolysis rate were also higher in unfiltered RBC compared to LR-RBC during storage. These observations pointed to possible involvement of leukocytes in more red cell membrane damage and a beneficial effect of leuko-reduction on the quality of stored RBC units. These differences may be related to bioactive substances such as cytokines and histamine released from leukocytes during storage, which implemented direct effects on RBC membrane and resulted in some structural and biochemical alterations^15^.

According to the suggestion of Castro and other researchers, the total hemoglobin concentration, lactate dehydrogenase and concentration of lactate are markers of hemolysis ^16^. 

Reduction in the number of leukocytes in RBC units diminished the damaging of ROS, whose production was be gained by the stimulation of NADP oxidase in leukocytes^17^.

Sonker et al. also reported that leuko-filtered RBC shows the lesser elevation of K^+^, LDH and hemolysis towards the end of storage period as compared to their unfiltered units. The presence of leukocytes may be the cause of enhanced oxidative stress. These authors observed that LDH enzyme activity, hemolysis, and cell membrane damage (potassium, LDH, free hemoglobin) enhanced in blood components with high lymphocyte count^18^.

Muller-Steinhardt et al. compared the filtered and unfiltered RBC units over a period of 42 days storage. They showed that hemolysis rates were initially higher in the filtered RBC units as compared to unfiltered RBC units in both groups. This selective depletion of damaged cells accounted for increased hemolysis immediately after filtration as seen in our study as well as in other studies. This process also was conversed until the end of the storage period. It means that in the final days of storage, hemolysis rate in unfiltered RBC was more than the LR-RBC. The level of hemolysis in all the units stays still well below the limit of 0.8% as proposed by the international guidelines^14^^, ^^19^.

Anaerobic glycolysis results in the increased concentration of lactic acid which may have caused a decrease in pH as observed in this study. Significant increase in LDH level may also be due to this reason. Hemolysis results in release of LDH in plasma and LDH best reflects the degree of hemolysis by its increased activity.

In our study, we observed a statistically significantly higher level of lactate and LDH in the two groups, but this rise in unfiltered RBC was more than the LR-RBC. These results have been confirmed by other investigators. Low values of hemolysis and LDH indicated less damage to the membranes of filtered RBC compared to unfiltered units. In relation to the lactate concentration, it can also be said that glycolysis was less in filtered bags.

Glucose-6-phosphate dehydrogenase (G6PD) is important enzyme in red blood cell metabolism and the key enzyme of the oxidative pentose phosphate pathway (PPP). In the PPP, nicotinamide adenine dinucleotide phosphate (NADP^+^) is converted into its reduced form NADPH, which is essential for GSH-mediated protection against oxidative stress^6^. 

Consumption of glucose leads to the reduction in the activity of the glycolytic pathway and reduction in G6PD during storage of RBC and consequently the decreased generation of NADPH. We assume that removed leukocytes from stored RBC cause stability of G6PD. We determined G6PD activity based on conversion of NADP into NADPH, and NADPH concentration was measured at 340 nm. The final calculation of enzyme activity was reported in the amount of hemoglobin (IU/g). The results of the current study also showed a decrease in G6PD activity during storage of both groups of RBC. Mean of G6PD activity on day of donation was 12.16 ± 29 IU/gHb in the two groups, whereas gradually decreased to 9.88 ± 0.27 and 10.48 ± 0.36 in RBC and LR-RBC after 42 days of storage, respectively (p-value = 0.001). There was a significant difference between the two groups and one group compared to the previous steps (p-value = 0.001). G6PD enzyme activity was less decreased in LR-RBC (9.88±0.27 IU/gHb) compared to unfiltered RBC (10.48±0.36 IU/gHb). These changes were statistically significant between the two groups (p-value=0.001), and were concurrent with changes previously observed by Peters et al. They showed that G6PD enzyme activity was 27.86 IU/L on *the third *day of *storage,* and gradually decreased to 18.06IU/L after 42 days of storage ^4^. 

Also, Ufelle et al. reported considerable decrease in G6PD activity in erythrocytes during storage from the third week of storage ^20^. 

Two studies concluded that G6PD function does not decrease significantly during the storage of RBCs ^21^^, ^^22^. In the analysis of Francis et al., the G6PD enzyme activity did not significantly change during 42 days of storage in RBC storage bags and attached segments^21^. These findings are in agreement with our results, whereas another study with similar methods reported the opposite results^23^.

According to the findings of our study, RBC indices such as MCV, MCH and MCHC were least affected by storage. The indices of MCH and MCHC were decreased during storage time, and the amount of MCV was increased in this period. These changes did not cause the significant differences between the two groups; however, there was a significant decrease in MCHC and MCH within one group on day 42 compared to day 35. Meanwhile, increased MCV showed that there was no significant difference between unfiltered RBC and LR-RBC. 

In this study, similar to Latham’s data, an elevation of MCV was noticed between days 35 and 42 and^12^. He found that MCV increased and MCHC decreased significantly during the storage period which were evaluated every week. These results are similar to our findings, whereas Baile et al. have indicated that MCHC remained basically unchanged and MCV decreased up to 28 days of storage^13^_._ Adias et al. showed that the amount of MCV was reduced, but MCH and MCHC increased during storage weeks. In overall, the results of these two studies are not consistent with our study^24^. This suggests that SAGM-stored RBCs have sufficient capacity and clearance mechanisms to decrease the effects of RCSL during storage.

## CONCLUSION

 In conclusion, this study and similar studies indicate that filtration with the use of additives solutions can be effective in improving the quality of stored red blood cells. Additional investigations on blood products can help dramatically enhance their quality and safety in blood banks.
